# Why do people overestimate the effectiveness of blocked learning?

**DOI:** 10.3758/s13423-022-02225-7

**Published:** 2022-12-16

**Authors:** Julia Krasnoff, Clara Overkott

**Affiliations:** Department of Psychology, Cognitive Psychology Unit, University of Zurich, Binzmuehlestrasse 14/22, 8050 Zurich, Switzerland

**Keywords:** Judgements of learning, Learning strategies, Metacognitive bias, Blocked learning

## Abstract

Interleaved learning has been shown to be better for delayed memory performance than blocked learning. Nevertheless, people judge blocked learning to be more effective. In the present work, we tested different explanations for this metacognitive bias. Across three experiments, participants studied sequences of object-color associations for a final color-reproduction test. In Experiment 1, colors of a sequence were selected from one color category (blocked-study condition) or distinct color categories (interleaved-study condition). Participants gave higher judgements of learning (JOLs) for objects studied in the blocked condition, although performance was better for objects in the interleaved condition. In Experiment 2, participants’ immediate memory performance after each sequence was additionally measured. JOLs were again higher for objects in the blocked condition, and they mimicked participants’ immediate memory performance suggesting a link between participants’ evaluations of the learning strategies and their immediate memory effects. In Experiment 3, the objects of one sequence were either grouped by category (blocked-study condition) or derived from distinct categories (interleaved-study condition). Neither JOLs, nor immediate performance was affected by this manipulation of blocked learning, speaking against the possibility that people prefer blocked learning because of habit only. We conclude that people overestimate the effectiveness of blocked learning due to the immediate memory boost caused by blocked learning and not due to their previously acquired habit to study in blocks. This study provides insights into how people evaluate the effectiveness of learning strategies and why these evaluations are not always accurate.

## Introduction

When a student prepares for a math test, they can either first practice addition and then proceed with subtraction or they can practice both mathematical operations in an interleaved way. Previous work has shown that interleaving different study material (also referred to as *interleaved learning*) is more beneficial for the learning outcome than studying related material in blocks, aka. *blocked learning* (e.g., Rohrer et al., 2014). This *interleaved-study benefit* has not only been found for practicing mathematical operations, but has been demonstrated for various study materials (for a meta-analysis, see Brunmair & Richter, 2019) and learning tasks, including category learning (e.g. Kornell & Bjork, 2008) and implicit motor sequence learning (e.g., Schorn & Knowlton, 2021). Despite this advantage of interleaved learning, people prefer studying material in blocks and additionally, judge blocked learning erroneously as more effective (Bjork et al., 2013). This overestimation of the effectiveness of blocked learning even persists after taking a test (Kornell & Bjork, 2008) and is difficult to mend (Yan et al., 2016). Consequently, people repeatedly choose this suboptimal learning strategy. The goal of the present study is contributing to a better understanding of the reasons for this detrimental, metacognitive bias.

To uncover the reasons for this metacognitive bias, we first focused on how people generally monitor their learning and make predictions about their future memory performance. Based on the predominant theory in the field – the cue-utilization approach – these predictions (aka. Judgements of Learning; JOLs) are made inferentially, based on a variety of cues (Koriat, 1997; Rhodes, 2016; Tauber & Rhodes, 2010). In contrast, the memory-strength theory for JOLs assumes that people predict their future performance by accessing their current memory representations (e.g., Krasnoff & Souza, 2021). Although, these two theories substantially differ in how they explain JOLs, they both assume that factors during learning (the available cues and the current memory strength for the representations, respectively) influence people’s predictions of their future memory performance. Focusing on those factors is therefore a promising approach to better understand people’s suboptimal preference for blocked learning.

Following this approach, we inspected the subsequent three hypotheses that have been previously discussed in context of the blocked learning-bias. The first explanation is what we refer to as *habit hypothesis* (Carvalho et al., 2016; Simon & Bjork, 2001; Tauber et al., 2013; Yan et al., 2016). Based on this hypothesis, people’s preference for blocked learning is acquired over the years: Already in school, pupils experience learning in blocks of different subjects and each subject is divided into blocks of related content (e.g., pupils first practice addition and only then subtraction). The constant early exposure to blocked learning leads to the belief that blocked learning is superior to interleaved learning. Consequently, people use the fact that material is presented in a block as a cue for their JOLs. The habit hypothesis therefore predicts that people always judge blocked learning as more effective than interleaved learning, regardless of any memory effects associated with those learning strategies.

The second explanation for this bias is the *immediate-boost hypothesis* that focuses on differences regarding the immediate memory effects caused by the two learning strategies (e.g., Schorn & Knowlton, 2021; Simon & Bjork, 2001): Blocked learning on the one hand is assumed to facilitate the learning process and thus to boost performance in the short-term. Interleaved learning, on the other hand, is assumed to be less beneficial in the short-term but to boost performance after some delay. The immediate-boost hypothesis builds on the idea that people evaluate learning strategies erroneously based on their immediate, instead of their long-term memory effects. Thus, this theory is consistent with the memory-strength theory for JOLs that assumes that people base their JOLs during learning erroneously on the strength of the representations stored in memory at that time point (Krasnoff & Souza, 2021). According to the immediate-boost hypothesis the explanation for the positive evaluation of blocked learning therefore lies in the fact that (1) people evaluate the effectiveness of learning strategies based on their immediate memory effect, and that (2) blocked learning boosts performance in the short-term. In contrast to the habit hypothesis, the immediate-boost hypothesis assumes that people solely judge blocked learning as more effective because it normally increases immediate memory performance. In situations in which immediate memory performance is not affected (or even harmed) by blocking material people should not show this bias.

Finally, the *fluency hypothesis* explains the blocked-learning preference by greater processing fluency in situations where study material is blocked. Because of the increased feeling of fluency during learning, people assume that learning is easy and that therefore their performance will be better (Birnbaum et al., 2013; Tauber et al., 2013; Wang & Xing, 2019). Although fluency is often reported as a cue for JOLs (e.g., Mueller et al., 2014; Undorf & Erdfelder, 2011; Undorf & Zimdahl, 2019), the mechanisms behind the formation of fluency are rarely specified in the context of blocked learning. This makes it difficult to derive concrete predictions from this hypothesis. For instance, it is possible that processing fluency is automatically increased when information is presented in a block in the study phase. In this case, the fluency hypothesis would make the same prediction as the habit hypothesis, namely that people will always judge blocked learning as more effective than interleaved learning. However, one could also assume that processing fluency is only greater when immediate memory is boosted through blocked learning. In this case, the fluency hypothesis would make the same predictions as the immediate-boost hypothesis, namely that people only judge blocked learning as more effective in situations where it boosts immediate memory performance. In contrast to the immediate-boost hypothesis, however, the blocked-learning bias would result from an increased feeling of fluency that is used as a cue for people’s judgements, and not from the immediate memory boost itself. Overall, the fluency hypothesis can make opposite predictions depending on the underlying processes one assumes.

To test these three hypotheses, we used an object-color binding task in which participants memorized the color of an object and reproduced it in a color-wheel when cued with the object at test (e.g., Prinzmetal et al., 1998; Souza et al., 2016; Zhang & Luck, 2008). We used this task because it enabled us to measure immediate memory performance and more precisely observe differences in performance between a blocked and an interleaved-study condition. Furthermore, the object-color task has not been used in the context of blocked versus interleaved learning yet and thus our study provides novel insights into the effects of these learning strategies on object-color binding learning. Finally, Sutterer and Awh (2016) have already used this task to study retrieval-practice showing that it is suitable to investigate the effects of learning strategies.

## Experiment 1

In the first step, we wanted to replicate the common findings on interleaved and blocked learning in the object-color task. Consistent with previous findings, we predicted that interleaved learning would lead to better memory performance than blocked learning. Furthermore, we expected that people would predict better performance in the blocked as compared to the interleaved-study condition.

### Method

#### Participants

As preregistered (https://osf.io/ket4p?view_only=2edb4b71379a4250a0a1103227970ec3), we collected a sample of 60 participants online on the platform Prolific. Only participants between 18 and 35 years, whose first language is English and who indicated to not have any visual impairment were recruited for this experiment. Participants were reimbursed with 2.50 GBP. We preregistered to exclude participants if they would not show any variance in JOLs. As all participants showed a variance in JOLs, the full sample was used for the analyses.

#### Materials and procedure

The experimental design was a within-subjects manipulation of the study method (interleaved vs. blocked study). The experiment was programmed in lab.js (Henninger et al., 2019). The main experimental task consisted of an object-color continuous reproduction task with a pool of 215 objects. For each participant, 98 objects were randomly selected to serve as memoranda in the experimental trials. Half of the objects were assigned to the interleaved condition and the other half to the blocked condition. Additionally, seven objects served for one practice trial.

**Fig. 1:**
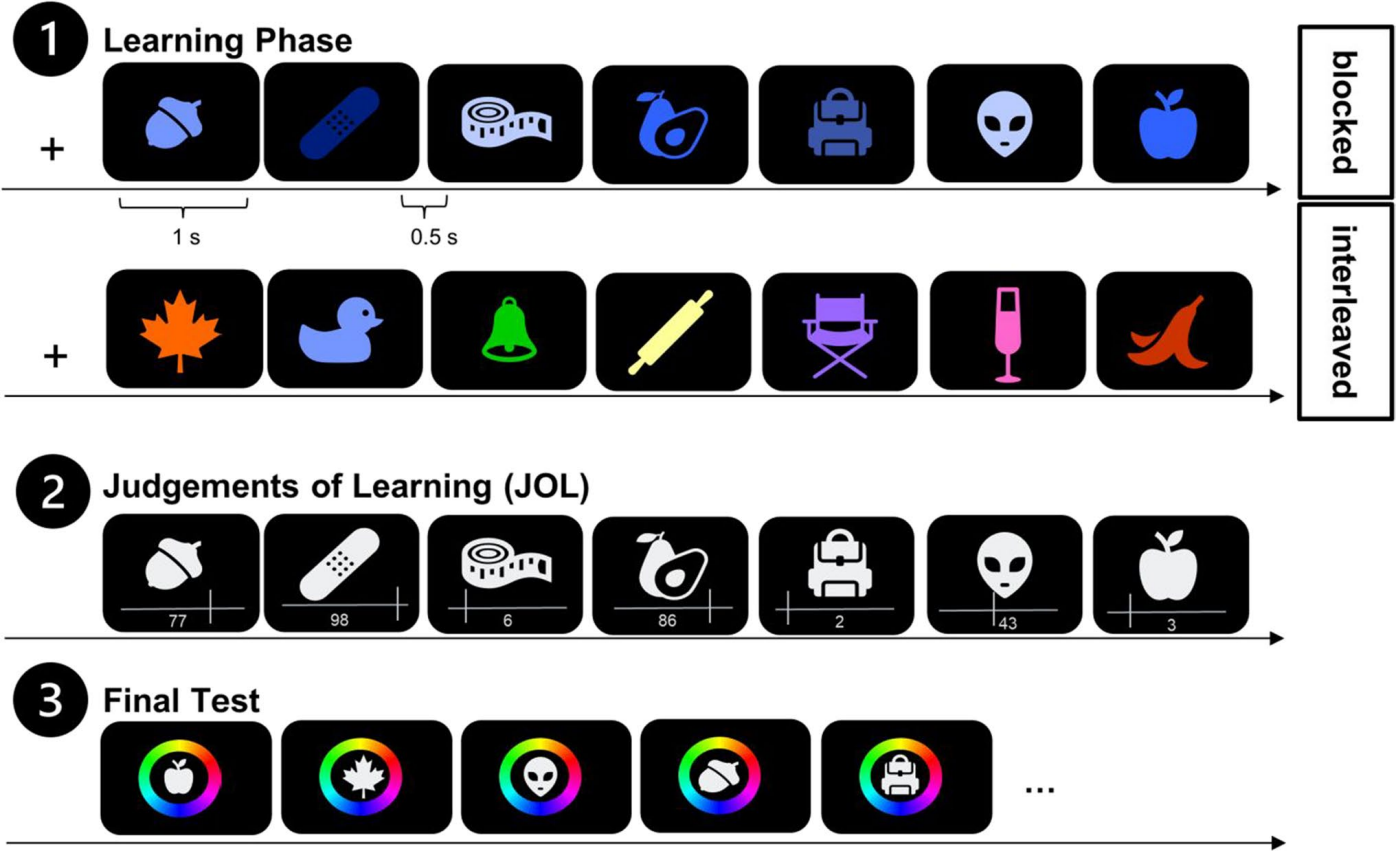
Experimental procedure of Experiment 1*.* Note. Flow of events of Experiment 1. The learning phase 1 is depicted for a blocked and interleaved study sequence. Phase 2 depicts the JOL phase that was the same for blocked and interleaved sequences. After learning and giving JOLs for 14 sequences, the final memory test followed in phase 3. In this final memory test, participants had to reproduce the color of all objects they learned in the interleaved and blocked study condition

The experimental procedure is depicted in Fig. 1. Participants completed a learning phase followed by a test phase. Each trial of the learning phase started with a light grey fixation cross that appeared in the middle of the screen. Participants started every trial with a mouse click. They were then presented with a sequence of seven colored objects. The colors were defined with the CIELAB color space with L = 70, a = 20, and b = 38, and radius = 60 (Zhang & Luck, 2008). Each colored object appeared in the middle of the screen against a black background for 1 s. Offset of the object was followed by a 0.5-s blank inter-stimulus interval before the next colored object was presented. In the blocked study condition, the colors of all objects of a sequence were drawn from one of the seven color categories (orange, pink, purple, blue, green, red, yellow). These seven color categories were identified in previous experiments (Overkott & Souza, 2021; Souza & Skóra, 2017) . Before drawing the colors, we first divided the 360 colors from the continuous color into these seven color categories (see Appendix Table 1 for more details). We then randomly selected seven different hues from one of the seven predefined areas (e.g., seven green hues).

In contrast to the blocked study condition, in the interleaved study condition the color of each object of a trial was randomly sampled from the seven distinct color categories (e.g., green, blue, orange, pink, red, yellow, purple). The order in which the conditions appeared was randomly shuffled for each participant. After the presentation of a sequence, participants’ JOLs were assessed. Therefore, the objects appeared again in serial order in a light grey color with a slider in white below and the mouse cursor. On the slider, participants were asked to rate how likely they will remember the color of the object in the color reproduction test in the end of the experiment. These JOLs were given on a continuous scale ranging from 1 (very poorly) to 100 (very well). The corresponding number appeared in white below the scale. The assessment of each JOL was followed by a 0.5-s blank.

Altogether, participants performed 14 trials (seven 7-object trials in the interleaved condition, and seven 7-object trials in the blocked condition), resulting in learning 98 colored objects overall. Thereby, participants studied 14 objects in each of the seven color categories (e.g., 14 yellow objects). Seven objects of a color category were studied in the interleaved and seven in the blocked study condition. All studied objects were then tested in a final test in random order. Each object appeared in grey surrounded by a color wheel (that randomly rotated for each object). Participants had to select the color they remembered for the depicted object from the color wheel. When moving the mouse cursor around the wheel, the object in the middle was filled with the color at the current position of the mouse cursor. When participants were satisfied with the selected color, they pressed the mouse button to confirm their response. After a delay of 0.5 s, the next object was probed. Prior to the start of the experiment, participants performed one practice trial to familiarize themselves with the task. The practice trial was excluded from the analysis.

After collecting data for all three experiments of this study, we found two minor coding errors in the experimental code. First, participants responses in the color-reproduction task were saved with an average deviation of 3° to the actual response that they selected. For example, if the participant wanted to click on the 350° angle, the registered response diverged from the actual intended response by ca. 3°. This error occurred throughout all tests and conditions and thus added to the measurement noise. Importantly, because all tests and conditions were similarly affected, this coding error did not bias any comparisons between the experimental conditions and thus is negligible for the interpretation of the results. Second, one hue of red (the hue at an angle of 0° that is located next to the pink color category; see Appendix Table 1) was accidently labeled as belonging to the red and pink color category. Consequently, this cue could appear in a red and likewise in a pink color sequence of the blocked study condition. However, because this is just one out of 360 hues, only 0.5% of the objects of Experiment 1 (and 0.6% of the objects of Experiment 2) were presented in this color. We calculated all analyses with and without these objects but did not observe any differences between the results. Therefore, we report the analyses of all trials here.

### Results

#### Effect of study condition on recall error in final test

**Fig. 2:**
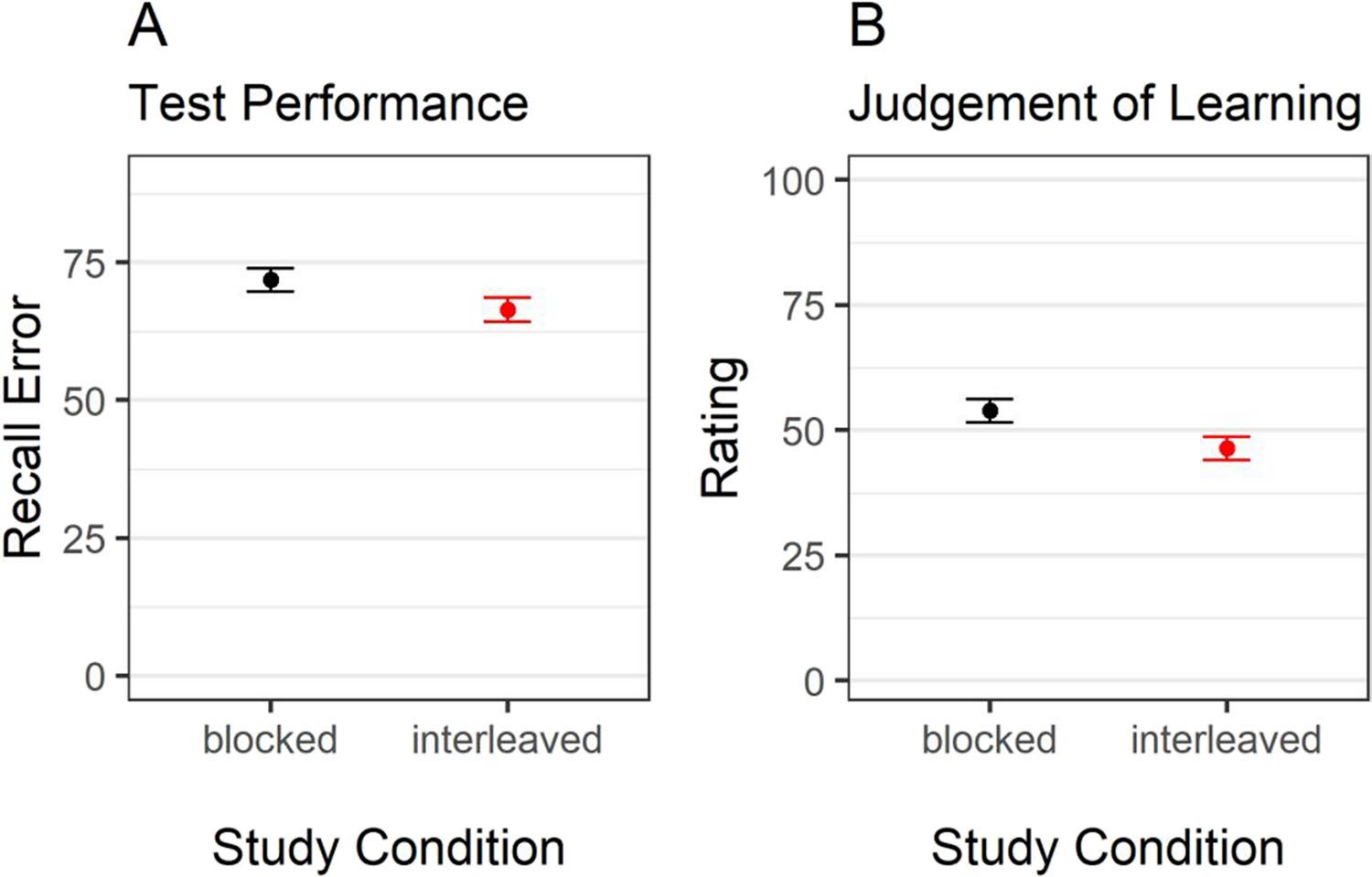
Recall error and judgements of learning as a function of study condition*.* Note. Error bars represent 95% within-participant confidence intervals

As preregistered, we first computed a measure of recall error for the final test data by computing the absolute difference between the presented color and participants’ response. This measure can range from 0° (the correct color was chosen) to 180° (a color on the opposite side of the color wheel was chosen). We then performed a Bayesian *t*-test for paired samples with study condition (blocked vs. interleaved) as predictor and recall error as criterion. The result provided evidence for an effect of study condition on recall error, BF_10_ = 34.51. As predicted, the recall error was higher for objects studied in the blocked as compared to the interleaved condition (see Fig. 2, Panel A), replicating the interleaved-study benefit.

#### Effect of study condition on judgements of learning

We then investigated participants’ JOLs by performing a Bayesian *t*-test for paired samples with study condition (blocked vs. interleaved) as predictor and JOLs as criterion. As predicted, JOLs were higher in the blocked than in the interleaved study condition, BF_10_ = 667.87 (see Fig. 2, Panel B).

Finally, we also computed an average Spearman correlation between JOLs and recall error by first computing a Spearman correlation coefficient for every participant. To obtain scores that follow a normal distribution, we transformed the correlation coefficients into Fisher’s *z*-values. Only then we computed the mean, which we then transformed back to Spearman’s *r* scale. As expected, participants’ JOLs only weakly correlated with their actual, predicted performance, *r* = -.13. A Bayesian one-sample *t*-test against *r* = 0 provided evidence in favor of this correlation, BF_10_ = 2.14 × 10^5^.

### Discussion

Experiment 1 provided a contextual replication of the interleaved-study benefit in a task that has not been used in this field yet, the object-color binding task. Consistent with previous literature, participants’ performance was better for objects presented in the interleaved as compared to the blocked-study condition. Despite this advantage, participants’ JOLs were higher for objects in the blocked-study condition. These findings are consistent with previous findings in the field and we therefore were able to use the color-object task for further assessing the different hypothesis regarding people’s blocked-learning bias in the following experiments.

## Experiment 2

To make conclusions regarding the immediate-boost hypothesis, we extended the procedure of Experiment 1 by including an immediate memory test after each sequence. The immediate-boost hypothesis assumes that people’s JOLs are based on immediate memory effects caused by the learning strategies. Thus, it predicts that immediate memory performance is better in the blocked as compared to the interleaved-study condition. Furthermore, it predicts that participants’ JOLs mimic participants’ immediate memory performance and thus are correlated with immediate recall error.

### Method

#### Participants

The data of this experiment was collected as part of a course for the Bachelor students at the University of Zurich. The course requirement for the students was to collect a minimum number of 75 participants. Overall, 77 participants were recruited for this experiment by the students of the course. Participants were between 18 and 35 years old and indicated to not have any visual impairment. Participants voluntary took part in this experiment or were reimbursed with course credit.

#### Materials and procedure

The design of this experiment was the same as in Experiment 1. Because this experiment consisted of more trials, overall, 210 objects were randomly selected to serve as memoranda in the experimental trials. Half of the objects were assigned to the interleaved condition, the other half to the blocked condition. Additionally, five objects served for one practice trial.

**Fig. 3:**
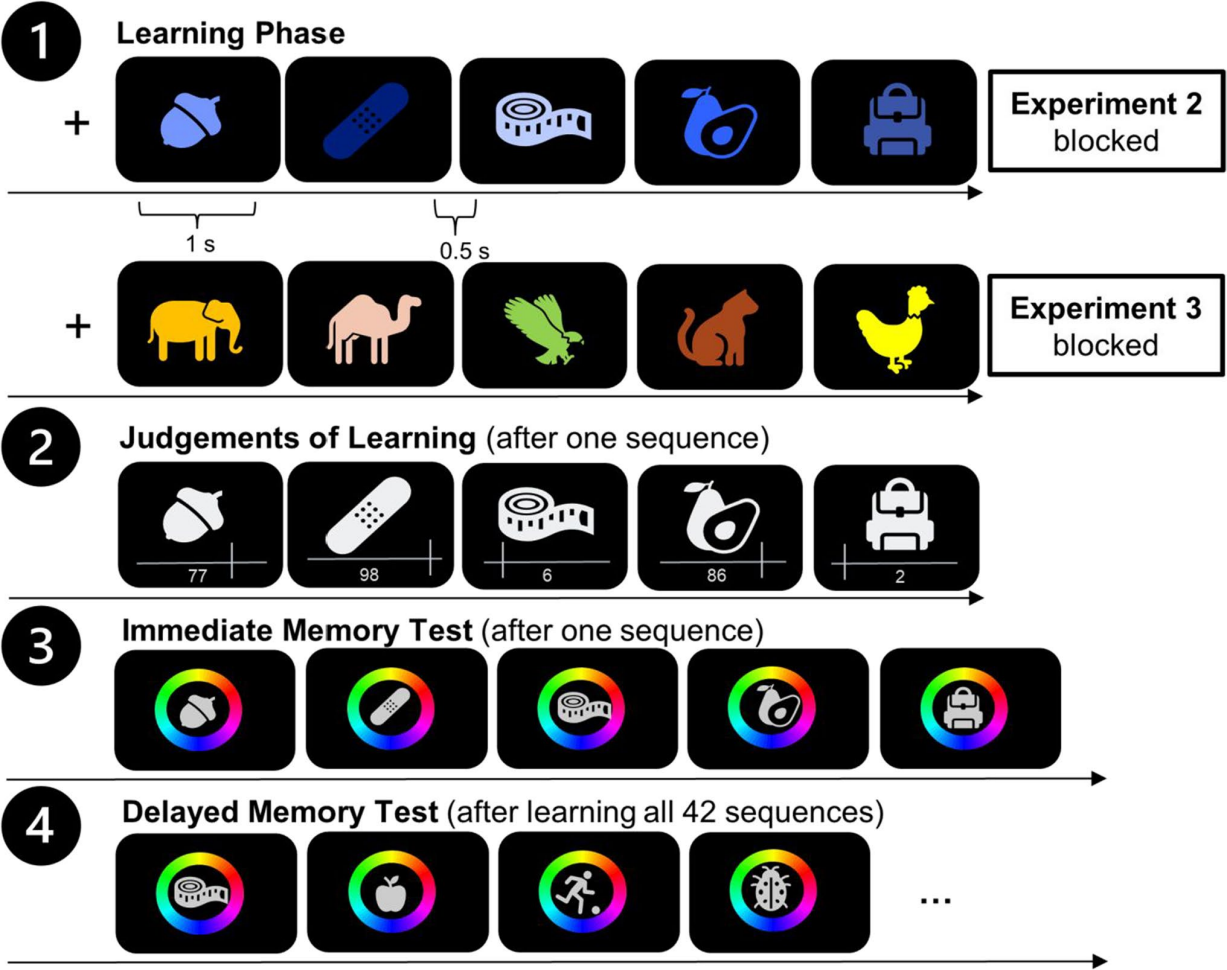
Experimental procedure of Experiments 2 and 3*.* Note. Flow of events of Experiments 2 and 3. The learning phase 1 is depicted for one blocked trial of Experiment 2 and in Experiment 3, respectively. Phase 2 depicts the JOL phase that was the same for blocked and interleaved sequences. Phase 3 depicts the immediate memory test. After learning the object-color associations, giving JOLs and performing an immediate memory test for all 42 sequences, the final memory test followed in phase 4. In this final memory test, participants had to reproduce the color of all objects they learned in the interleaved and blocked study condition

The experimental procedure solely differed from Experiment 1 regarding the following aspects: The first modification was that participants were presented with sequences of five instead of seven colored objects (see Fig. 3). In the blocked-study condition, colors of all objects of a sequence were drawn from one of the seven color categories. In the interleaved study condition the color of each object of a trial was randomly sampled from five of the seven distinct color categories (e.g., green, blue, orange, pink, red). Again, the order of conditions was randomly shuffled for each participant. The second modification was that we assessed participants’ immediate memory performance after participants gave their JOLs. Therefore, the objects appeared in serial order and participants had to reproduce the color of the objects on the color wheel.

Altogether, participants performed 42 trials (21 five-object trials in the interleaved condition, and 21 five-object trials in the blocked condition), resulting in learning 210 colored objects overall. Thereby, participants studied 30 objects in each of the seven color categories (e.g., 30 yellow objects). Fifteen objects of a color category were studied in the interleaved and 15 in the blocked learning condition. Again, all studied objects were tested in a final test in random order. Prior to the start of the experiment, participants performed one practice trial to get familiar with the task. The practice trial was excluded from the analysis.

### Results

#### Effect of study condition on recall error in immediate and final test

**Fig. 4:**
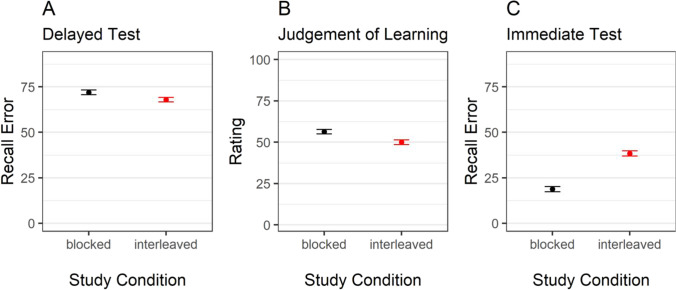
Recall error in the immediate and delayed test and judgements of learning as a function of study condition*.* Note. Error bars represent 95% within-participant confidence intervals

We first performed a Bayesian ANOVA with test time point and study condition as predictors, recall error as predicted variable, and participants as random effect. We conducted this analysis in R (R Core Team, 2018) using the default prior settings of the BayesFactor package (Morey et al., 2018). The results provided overwhelming evidence in favor of an interaction between study condition and test time-point on recall error, BF_10_ = 2.05 × 10^122^. We then performed two Bayesian *t*-tests for paired samples with study condition (blocked vs. interleaved) as predictor and recall error in the immediate and final test, respectively, as criterion. As in Experiment 1, the recall error in the delayed test was higher in the blocked as compared to the interleaved-study condition with a BF_10_ = 500.58. In contrast, but in line with immediate-boost hypothesis, the recall error in the immediate test was higher for objects studied in the interleaved as compared to the blocked condition, BF_10_ = 4.14 × 10^27^ (see Fig. 4, Panel C). Whilst blocked learning led to poorer performance in the final test, it boosted performance in the immediate test.

#### Effect of study condition on judgements of learning

We then performed a Bayesian *t*-test for paired samples with study condition as predictor and JOLs as criterion. Again, JOLs were higher in the blocked as compared to the interleaved-study condition, BF_10_ = 8.94 × 10^5^ (see Fig. 4, Panel B), replicating the results of Experiment 1.

Finally, we computed an average Spearman correlation between JOLs and recall error in the immediate and final test. We followed the same procedure as in Experiment 1. Again, JOLs only weakly correlated with final test performance, *r* = - .08 (BF_10_ = 6.8 × 10^5^), indicating poor JOLs’ accuracy. JOLs and immediate recall showed a small to moderate correlation, *r* = - .17 (BF_10_ = 1.02 × 10^16^). A Bayesian *t*-test for paired samples revealed that JOLs were higher correlated with immediate than delayed recall error with BF_10_ = 7.41 × 10^3^.

### Discussion

By investigating immediate memory effects, we showed that participants’ immediate performance was credibly better in the blocked as compared to the interleaved condition and that participants’ JOLs mimicked this performance. This is in line with the idea that the blocked-learning bias results from a beneficial effect that blocking material has on immediate memory performance.

What does this finding tell us regarding the three different hypotheses? This finding is directly predicted by the immediate-boost hypothesis that explains the overestimation of blocked learning by its immediate memory effect. It can also be accommodated by the version of the fluency hypothesis that assumes that the increase in processing fluency in the blocked-study condition directly results from an immediate memory boost. Even though, these findings show that people’s evaluation of the blocked-study condition is directly linked to its immediate memory effects, they do not discard the habit hypothesis: It is well possible that people prefer blocked learning because of both, its immediate-memory boost, and their habit. To be able to differentiate between these hypotheses, we conducted Experiment 3.

## Experiment 3

To create a situation in which the immediate-boost hypothesis and the habit hypothesis make different predictions, we implemented a manipulation of blocked and interleaved-study in which blocked-study does not boost immediate memory performance. Instead of blocking the colors of a sequence, we blocked the objects of a sequence: We presented either examples of a single category (e.g., animals) in the blocked-study condition or examples of five different categories in the interleaved-study condition. The colors of the objects were randomly sampled in both conditions. We assumed that immediate performance will be negatively affected by blocking the objects by categories. This assumption was derived from studies on different forms of similarity that show that similarity leads to more confusions in working memory (Ishiguro & Saito, 2021; Jalbert et al., 2008). We expected that participants will confuse the objects more often with each other when they are sampled from one category. In contrast, when objects are sampled from different categories those confusions should occur less often. Consequently, we predicted that – in contrast to Experiment 2 – the immediate recall error is higher in the blocked than interleaved-study condition.

By implementing this form of blocked learning, we created a situation in which the three hypotheses make different predictions regarding people’s JOLs: The habit hypothesis predicts higher JOLs in the blocked-study condition regardless of its immediate memory effect. In contrast, the immediate-boost hypothesis predicts that JOLs follow immediate memory performance and thus will not be higher in this operationalization of blocked-learning. As already pointed out, the predictions of the fluency hypothesis are less straightforward. Depending on what is assumed to cause the increase in fluency in the blocked-learning condition, this hypothesis would either make the same prediction as the habit hypothesis (assuming that fluency is increased automatically when study material is blocked) or the immediate-boost hypothesis (assuming that fluency is solely increased due to an immediate memory boost).

### Method

#### Participants

The data of this experiment was also collected as part of a course for the Bachelor students at the University of Zurich. The course requirement was to collect a minimum number of 75 participants. Overall, 85 participants were recruited for this experiment by the Bachelor students. Participants were between 18 and 35 years old and indicated to not have any visual impairment. Participants voluntary took part in this experiment or in exchange for course credit.

#### Materials and procedure

The design and the procedure of this experiment was equal to Experiment 2. The only difference was the operationalization of interleaved versus blocked study and thus, the study material. As in Experiment 2, participants studied sequences of five colored objects. However, in this experiment the study method was operationalized by presenting either five objects from the same category in the blocked-study condition (e.g., five animals) or five objects from different categories in the interleaved-study condition (e.g., an animal, a piece of clothes, a tool, a fruit, a music instrument). In contrast to the two previous experiments, the colors of the objects were randomly sampled from the 360 colors of the color wheel in both within-subject study conditions. To keep everything else equal to Experiment 2, the objects were sampled from overall seven different categories. Those categories were animals, plants, clothes, tools, music instruments, fruit and vegetables, and sports. As in Experiment 2, participants overall studied 30 objects from each of the seven categories (e.g., 30 animals). Fifteen objects of a category were studied in the interleaved and 15 in the blocked learning condition. As in the previous experiments, participants performed one practice trial to get familiar with the task. Data from the practice trial was excluded from the analysis.

### Results

#### Effect of study condition on recall error in immediate and final test

**Fig. 5:**
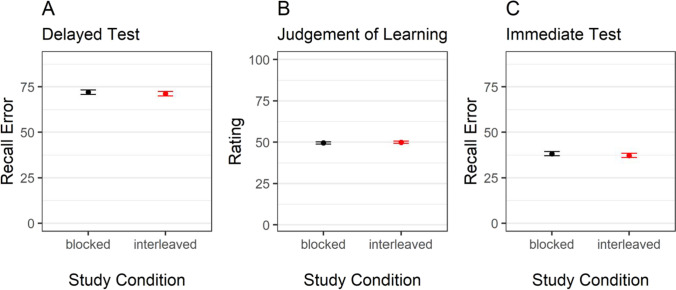
Recall error in the immediate and delayed test and judgements of learning as a function of study condition*.* Note. Error bars represent 95% within-participant confidence intervals

The results of a Bayesian ANOVA with test time point and study condition as predictors, recall error as criterion, and participants as random effects provided anecdotal evidence against an interaction between study condition and test time-point on recall error, BF_10_ = 0.34. We further performed two Bayesian *t*-tests for paired samples with study condition as predictor and recall error in the immediate and final test, respectively, as criteria. The results are depicted in Fig. 5.

In contrast to Experiments 1 and 2, recall error in the delayed test did not differ between the two study conditions with BF_10_ = 0.16. The same applied for the recall error in the immediate test where – against our prediction – we found evidence against an effect of study condition with BF_10_ = 0.24 (see Fig. 4, Panel C).

#### Effect of study condition on judgements of learning

We performed a Bayesian *t*-test for paired samples with study condition (blocked vs. interleaved) as predictor and JOLs as criteria. In contrast to Experiments 1 and 2, JOLs also did not differ between the study conditions, BF_10_ = 0.16 (see Fig. 5, Panel B).

JOLs correlated with final test performance with *r* = - .14 (BF_10_ = 1.42 × 10^11^). JOLs and immediate recall moderately correlated with *r* = - .23, (BF_10_ = 2.71 × 10^20^). As in Experiment 2, JOLs were credibly higher correlated with immediate than delayed recall error (BF_10_ = 9.37 × 10^6^), showing that participants relied on immediate memory effects when making their JOLs.

### Discussion

In this experiment, we implemented a manipulation of blocked learning that did not affect immediate memory performance. Against the prediction of the habit hypothesis, here we did not find a difference in JOLs between the two study conditions. Thus, we can discard the idea that people overestimate the effectiveness of blocked learning solely because of an acquired habit. The finding also challenges the version of the fluency hypothesis that assumes that processing fluency is automatically increased by blocking material. If this was the case and people used this feeling of fluency to evaluate the learning strategies, we would have expected to find higher JOLs in the blocked-study condition, which we did not observe.

Against our prediction, the manipulation of the study condition did not lead to poorer immediate performance in the blocked condition: The mean recall error was the same for sequences with objects from one category as compared to sequences with objects from five categories. Although we expected worse immediate performance in the blocked-study condition, this finding seems consistent with the literature on semantic similarity: In contrast to other forms of similarity, semantic similarity does not (consistently) lead to an increased rate of confusion errors in memory tasks (Saint-Aubin & Poirier, 1999). Even though we did not expect this finding, it does not change the conclusion regarding the immediate-boost hypothesis. Again, we found that JOLs mimic immediate memory performance and that JOLs and immediate recall error are correlated. Thus, the results provide further evidence for the immediate-boost hypothesis. The results are also in line with the version of the fluency hypothesis that assumes that people overestimate the effectiveness of blocked learning because of the perceived feeling of fluency that occurs due to an immediate memory boost in the blocked-study conditions. Overall, people seem to overestimate the effectiveness of blocked learning when it boosts immediate memory performance. However, when it does not influence immediate memory performance, people do not show this metacognitive bias.

## General discussion

In many situations, interleaving study material when learning leads to better performance than studying material in blocks. Nevertheless, people strongly believe that blocked learning is more effective. In the present study, we replicated and expanded these effects to an experimental procedure that has not been used yet – an object-color binding task. We further found that the overestimation of the effectiveness of blocked learning is due to an immediate memory boost caused by this study method. This finding rules out the habit hypothesis, postulating that people solely overestimate the effectiveness of blocked learning due to repeated exposure to this learning strategy. It also challenges the version of the fluency hypothesis that assumes that blocking material automatically leads to an increase in fluency and thus, to an overestimation of the effectiveness of this learning strategy. In contrast, the findings provide clear evidence for the immediate-boost hypothesis that explains the blocked-learning bias by a short-term memory advantage of blocked over interleaved learning.

Even though our data speaks against the habit explanation and in favor of the immediate-boost hypothesis, it does not allow for strong conclusions regarding the fluency hypothesis. This is because, based on our data, we cannot distinguish between a direct and a mediated effect of the immediate memory effects on JOLs. More concretely, it is well possible that the immediate-memory boost leads to an increase in processing fluency and that people use the feeling of fluency as a cue for their JOLs, consequently overestimating the effectiveness of blocked learning. An important direction for future studies will be to distinguish between this version of the fluency hypothesis and the immediate-boost hypothesis.

Despite this limitation, the present study clearly suggests that people evaluate the effectiveness of learning strategies not based on their habit, but rather (directly or indirectly) based on how much they boost their memory in the short-term. This way of evaluating a learning strategy is problematic given that strategies that are effective in the long-term are not necessarily the ones that boost performance in the short-term. This not only holds for interleaved learning but also for many other effective strategies (so-called *desirable difficulties*; Bjork, 2019), such as elaboration or retrieval-practice that do either not boost immediate performance or even slow down the learning process at first (Bartsch & Oberauer, 2021; Roediger & Karpicke, 2006). To improve self-regulated learning, future research should therefore focus on finding ways to dissociate people’s evaluations of learning strategies from their (oftentimes) undiagnostic immediate memory effects.

## Appendix

Appendix Table 1

**Table 1:** Color categories in the color wheel

	First Color	Last Color
Red	0	25
Orange	26	96
Yellow	97	120
Green	121	205
Blue	206	256
Purple	257	286
Pink	287	359
